# A Predictive MRI Radiomics Model for Histologic Differentiation in Soft Tissue Sarcomas

**DOI:** 10.3390/cancers18101667

**Published:** 2026-05-21

**Authors:** Laetitia Perronne, Nicolò Gennaro, Zuzanna Kobus, Mirinae Seo, Amir A. Borhani, Linda Kelahan, Hatice Savas, Ryan Avery, Kamal Subedi, Chase Krumpelman, Gorkem Durak, Ulas Bagci, Akhil Chawla, Borislav Alexiev, Pedro Hermida de Viveiros, Seth Pollack, Yuri S. Velichko

**Affiliations:** 1 Department of Radiology, Northwestern University Feinberg School of Medicine, Chicago, IL 60611, USAsmilkys81@gmail.com (M.S.); linda.kelahan@nm.org (L.K.);; 2 Robert H. Lurie Comprehensive Cancer Center, Northwestern University Feinberg School of Medicine, Chicago, IL 60611, USAseth.pollack@northwestern.edu (S.P.); 3 Department of Surgery, Northwestern University Feinberg School of Medicine, Chicago, IL 60611, USA; 4 Department of Pathology, Northwestern University Feinberg School of Medicine, Chicago, IL 60611, USA; 5 Department of Medicine, Northwestern University Feinberg School of Medicine, Chicago, IL 60611, USA

**Keywords:** soft tissue sarcoma, MRI, radiomics, histologic differentiation

## Abstract

**Background/Objectives:** The aim of this study was to develop and validate a robust, radiomics-based classification model that uses pre-treatment MRI to non-invasively differentiate among major soft tissue sarcoma (STS) subtypes and a benign mimic. **Methods:** In this retrospective study, a cohort of 332 patients with biopsy-proven leiomyosarcoma, myxofibrosarcoma, myxoid liposarcoma, dedifferentiated liposarcoma, and undifferentiated pleomorphic sarcoma, along with the benign mimic intramuscular myxoma, was analyzed. Pre-treatment T1-weighted fat-saturated contrast-enhanced and T2-weighted fat-saturated MRI sequences were used for analysis. Following manual tumor segmentation, 1240 three-dimensional radiomic features were extracted. An XGBoost classifier was trained and validated using a robust 250-iteration bootstrap framework with nested cross-validation to ensure rigorous feature selection and unbiased performance evaluation. The model’s performance was assessed independently on T1-only, T2-only, and combined T1+T2 feature sets. **Results:** The combined T1 and T2 model achieved superior performance with an accuracy of 0.68 ± 0.04 and an AUC of 0.92 ± 0.02. At the subtype level, balanced accuracy was highest for intramuscular myxoma (0.91 ± 0.05), dedifferentiated liposarcoma (0.84 ± 0.06), and leiomyosarcoma (0.83 ± 0.05). SHAP analysis identified key features driving predictions, such as low T2 GLSZM Zone Size Entropy for myxoma and high T2 GLSZM Gray-Level Variance for leiomyosarcoma, which aligns with known pathological characteristics. Misclassifications predominantly occurred between subtypes with overlapping radiomic profiles. **Conclusions:** Radiomics applied to pre-treatment MRI enables robust, non-invasive classification of STS subtypes, demonstrating strong clinical potential for improving diagnostic confidence and informing triage strategies.

## Simple Summary

Soft tissue sarcomas are rare, complex cancers that are difficult to diagnose accurately. While biopsies are the standard tool, they are invasive and can be inaccurate because these tumors are often inconsistent throughout their structure. Standard medical imaging also frequently fails to distinguish between different cancer subtypes. This research aimed to develop a non-invasive computer model that analyzes hidden data within MRI scans to identify five major sarcoma types and one benign condition that mimics cancer. Our results demonstrate that this approach can reliably classify these tumors, offering a powerful tool to improve diagnostic confidence. By providing a clearer picture of the specific cancer type without additional invasive procedures, this technology helps doctors personalize treatment plans and provides a foundation for future advancements in cancer imaging and research.

## 1. Introduction

Soft tissue sarcomas (STS) are rare and highly heterogeneous mesenchymal tumors, accounting for less than 1% of adult malignancies but representing over 75% of all sarcomas, with more than 13,000 new diagnoses annually in the United States [1,2,3,4]. They arise in various anatomical locations and encompass a wide spectrum of histopathological and genetic subtypes, contributing to significant diagnostic and research challenges [5,6]. Although biopsy with histopathological evaluation remains the gold standard for diagnosis [5], it is invasive and may be affected by sampling errors, particularly in heterogeneous or difficult-to-access tumors [7,8]. In addition, misclassification may occur in non-specialized settings lacking sarcoma expertise [9]. MRI plays a central role in the non-invasive assessment of STS, providing detailed information on tumor extent and its relationship to surrounding structures [10,11]. However, qualitative MRI interpretation alone is generally insufficient for reliable subtype differentiation, as many sarcomas exhibit overlapping signal characteristics, enhancement patterns, and morphological features, limiting the ability of imaging to replace histologic diagnosis for pre-biopsy subtype prediction [12]. In clinical practice, this limitation frequently leads to diagnostic uncertainty when evaluating soft tissue masses prior to biopsy. Radiologists are often unable to confidently distinguish between histologic subtypes or even between benign and malignant lesions based on imaging alone. This uncertainty may affect biopsy targeting, delay appropriate treatment decisions, and in some cases lead to suboptimal sampling of heterogeneous tumors.

To address these challenges, radiomics has emerged as a promising quantitative imaging strategy. It extracts vast amounts of quantitative data from standard medical images, many imperceptible to the human eye [13,14,15]. These features can capture intratumoral heterogeneity and complex spatial patterns that may reflect underlying tumor biology. When combined with machine learning algorithms, they can create predictive models for tumor classification and outcome prediction [16,17]. Early studies have demonstrated the utility of radiomics in sarcoma characterization, such as discriminating tumor grades, predicting responses to neoadjuvant therapy, and assessing metastatic potential [18,19,20,21,22,23,24,25,26]. Despite this progress, the development of clinically impactful STS imaging models is hindered by a crucial gap: a lack of subtype-specific analyses, as many existing studies have grouped various STS subtypes together, and the challenge of developing robust models suitable for the small datasets inherent to rare tumor research. The direct application of radiomics for histologic subtype classification remains significantly underexplored. Given the critical importance of accurate subtype identification in sarcoma management, this represents a significant opportunity for research and innovation.

To address this specific gap, this study aimed to develop and validate a radiomics-based classification model using pre-treatment MRI data to distinguish among major STS subtypes and a benign mimic. We focused on six diagnostically challenging and clinically relevant categories: leiomyosarcoma, myxoid liposarcoma, dedifferentiated liposarcoma, undifferentiated pleomorphic sarcoma, myxofibrosarcoma, representing over 50% of all adult soft tissue sarcomas, and the benign intramuscular myxoma, the latter to assess the model’s capacity for malignant versus benign differentiation.

## 2. Materials and Methods

### 2.1. Study Population

**Figure 1:**
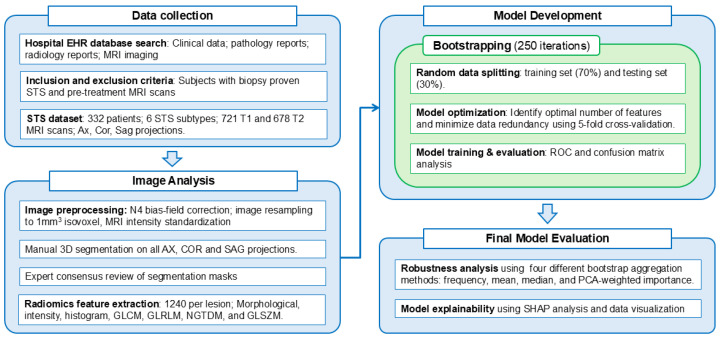
Schematic illustration of the methodology for developing a radiomics-based model to classify STS subtypes.

This retrospective study received Institutional Review Board (IRB) approval. According to the Northwestern University IRB, informed consent was waived. We identified a patient cohort by searching the electronic data warehouse (EDW) of a single, multi-hospital health system between January 2004 and December 2022 for individuals with biopsy-proven STS who had a pre-treatment MRI of the primary tumor. After the EDW search, anonymized MRI scans were retrieved from the institutional picture archiving and communication system (PACS). Patients were eligible if at least one of these MRI sequences was available: T1-weighted fat-saturated contrast-enhanced (T1 FS CE) and T2-weighted fat-saturated sequences (T2 FS), acquired in axial, coronal, and sagittal planes using standard clinical protocols (see Supplemental Materials, Table S1). Figure 1 provides a summary of the study workflow.

**Figure 2:**
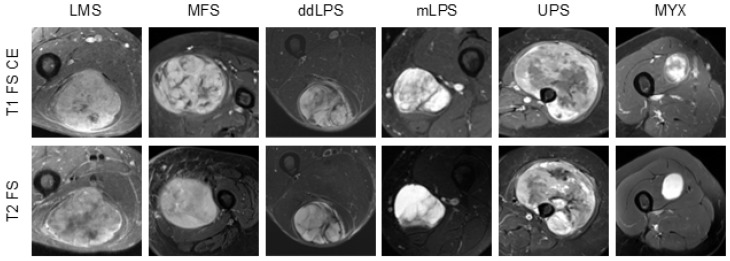
Representative T1-weighted fat-saturated contrast-enhanced and T2-weighted fat-saturated MRI images of six different STS subtypes: Leiomyosarcoma (LMS), Myxofibrosarcoma (MFS), Dedifferentiated Liposarcoma (ddLPS), Myxoid Liposarcoma (mLPS), Undifferentiated Pleomorphic Sarcoma (UPS), and Myxoma (MYX).

Among 780 initially identified patients, we selected cases meeting inclusion criteria and focused on six diagnostically challenging and clinically relevant tumor subtypes with sufficient representation for model development: leiomyosarcoma (LMS), myxofibrosarcoma (MFS), myxoid liposarcoma (mLPS), dedifferentiated liposarcoma (ddLPS), undifferentiated pleomorphic sarcoma (UPS), and intramuscular myxoma (MYX). All included diagnoses were confirmed by dedicated bone and soft tissue pathologists at our sarcoma referral center. Figure 2 displays sample T1-weighted and T2-weighted fat-saturated MRI images for the analyzed subtypes. A subset of this dataset was previously utilized for tumor segmentation and the assessment of response to neoadjuvant therapy [27,28,29]. A detailed summary of the dataset, including subtype distribution, demographic characteristics, and imaging parameters, is provided in Section 3 (Table 1). Detailed MRI acquisition parameters, including echo time (TE), repetition time (TR), pixel spacing, and slice thickness for both T1 and T2 sequences across all six STS subtypes, are provided in Supplementary Materials, Table S1.

### 2.2. Tumor Segmentation and Radiomic Feature Extraction

Individual lesions were segmented volumetrically from the available T1-weighted post-contrast fat-saturated and T2-weighted fat-saturated MRI projections using LIFEx (version 7.6, Orsay, France) [30]. To mitigate bias, a team of trained radiologists (L.P., N.G., and M.S.) and a fourth-year medical student (Z.K.) performed the segmentations while blinded to the histopathologic diagnoses. L.P. and N.G. then reviewed these segmentations for quality and accuracy. N.G. was the only reader unblinded during this process, having previously collected the pathology results. To quantitatively assess inter-observer variability, a subset comprising approximately 20% of the dataset was segmented by two readers (N.G. and L.P.). Inter-reader reliability was evaluated using the intra-class correlation coefficient (ICC) [31,32].

Each MRI sequence (axial, coronal, or sagittal) consisted of a multi-slice volumetric acquisition. Not all imaging planes were available for every tumor (see details in Supplementary Materials, Paragraph S1). When available, tumors were segmented volumetrically on each sequence independently. Radiomic features were subsequently extracted from each segmented volume and each MRI sequence using relative intensity rescaling (mean ± 3 standard deviations) and 32 gray-level binning. Radiomic features were categorized into three main groups: The first is the shape (morphology) of radiomic features; then, first-order intensity radiomic features computed from voxel intensity distributions within the segmented volume; and finally, higher-order texture radiomic features extracted from gray-level co-occurrence (GLCM), gray-level run-length (GLRLM), gray-level size zone (GLSZM), and neighboring gray tone difference (NGTDM) matrices [30]. For each available volumetric plane, the full set of radiomic features was extracted independently. To capture intratumoral heterogeneity across various spatial scales and ensure adequate sampling of small lesions, GLCM radiomic features were calculated for distances ranging from 1 to 5 mm. In total, 1240 3D radiomic features were computed per lesion across the available MRI sequences.

### 2.3. Handling of Missing Radiomic Data

For subjects with missing MRI sequences, a linear mixed-effects model (LME) was used to impute missing radiomic features [33,34,35,36]. This approach leveraged the association between radiomic features and tumor volume, while treating STS type as a fixed-effect variable to account for their potential confounding effects. Specifically, missing T1- or T2-based radiomic features were calculated directly from the resulting LME fitting equation, using the subject’s tumor volume and STS subtype (treated as a fixed effect to control for confounding) as inputs. To prevent data leakage, the LME model parameters were calculated using only the training partition of each split and were subsequently applied to the corresponding test set. Figure S1 (Supplemental Materials) demonstrates the association between both T1-weighted and T2-weighted radiomic features and tumor volume for all STS subtypes.

### 2.4. Machine Learning Workflow and Statistical Analysis Overall Experimental Design

Our framework was designed to address the challenges of tumor heterogeneity and small sample sizes inherent to STS. We employed a bootstrap resampling strategy to develop robust models and identify a stable feature signature. To assess the contribution of different imaging data, three separate XGBoost classifiers were developed and evaluated: one using T1-weighted radiomic features alone, one using T2-weighted radiomic features alone, and one combining radiomic features from both MRI sequences. XGBoost was selected due to its robustness in high-dimensional settings, its ability to model non-linear relationships, and its regularization capabilities, which help reduce overfitting in relatively small datasets. The machine learning workflow consisted of two main stages. The first was robust feature selection within a bootstrap framework using nested cross-validation and performance estimation of the bootstrap-specific models on independent test splits. The second was construction and interpretation of a Final Robust Model using the feature signature.

### 2.5. Bootstrap Framework for Feature Selection

An XGBoost classifier was trained within a bootstrap framework (250 iterations) to ensure robust feature selection and performance evaluation. In each iteration, the dataset was randomly split into a 70/30 train/test set, stratified by subtype. A two-stage feature selection procedure, governed by nested 5-fold cross-validation (NCV), was then applied exclusively to the 70% training data. This NCV procedure identified the optimal number of radiomic features (*N_opt_*) by testing feature sets of varying sizes (*N* = 10 to 300), ranking radiomic features by importance, and filtering for multicollinearity (pairwise correlation > 0.90). The quality of the resulting filtered feature subset was evaluated on the held-out validation set of the outer fold. After this was repeated for all outer folds and all candidate values of *N*, the optimal feature count, *N_opt_*, was determined as the *N* that yielded the best average performance across the outer folds. The resulting final feature set, the model performance, and its importance scores were saved, concluding the process for that single bootstrap iteration. In this context, the importance score represents the average gain, which measures a feature’s overall contribution to improving the model’s predictive accuracy.

For each bootstrap iteration, performance metrics were computed on the independent test split. To evaluate subtype-specific performance, a one-vs-rest (OvR) approach was utilized to generate subtype-specific Receiver Operating Characteristic (ROC) curves. To assess global discriminative performance, macro-average and micro-average ROC curves were also calculated. For each iteration, sensitivity values were interpolated onto a fixed standard grid of specificities to enable consistent aggregation. The macro-average ROC curve was computed using the unweighted mean of the interpolated sensitivities across all six STS subtypes, ensuring equal representation regardless of class prevalence. The micro-average ROC curve was generated by aggregating the true binary labels and predicted probabilities from all classes into a single evaluation array, effectively weighting performance by class frequency. Finally, these metrics and curves were aggregated across all 250 iterations to estimate the overall distribution and expected performance—including accuracy, macro-averaged AUC, multiclass Cohen’s kappa, and mean ROC curves with 95% confidence intervals—of the bootstrap-specific models.

Overall, this multi-step feature selection strategy was specifically designed to reduce redundancy among radiomic features and limit overfitting by retaining only stable and reproducible radiomic features across multiple resampled datasets.

### 2.6. Identification of a Globally Robust Feature Signature

**Figure 3:**
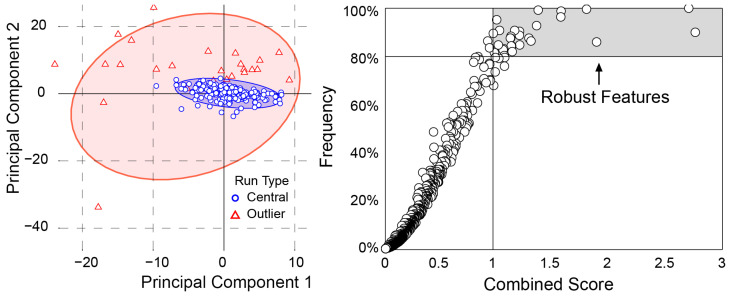
Robustness analysis of radiomic features. (**Left**) PCA plot demonstrating the consistency of the model; the tight cluster of blue circles indicates that most of the 250 bootstrap iterations produced highly similar results. (**Right**) Selection frequency of radiomics features plotted against their predictive strength (Combined Score). Features in the top-right shaded region (frequency > 80%) represent the most robust and reliable markers selected for the Final Robust Model.

Following 250 iterations, the resulting collection of unique feature sets was aggregated to distill a single, globally robust predictive signature. This robustness analysis involved ranking all radiomic features that appeared in any of the sets using four statistical metrics: (1) feature frequency, the total number of times each feature was selected; (2) mean importance, the average importance score across all runs; (3) median importance, a measure robust to outliers; and (4) PCA-weighted importance. To determine this PCA-weighted importance, we first applied PCA to the matrix of feature importance scores across all runs. We then represented each bootstrap iteration as a point in this PCA space (Figure 3) and calculated its distance from the centroid, assigning higher weights to runs closer to the center before computing an average of each feature’s importance.

The final feature selection process applied a Stability Filter (frequency > 80%) and a Signal-to-Noise Filter (Combined Score > 1). The Combined Score for each radiomic feature was calculated as the ratio of its mean importance to its standard deviation. This dual approach ensures that each selected feature is not only consistently relevant across data samples, but also has an average importance that significantly outweighs its statistical variability.

### 2.7. Final Robust Model

A final XGBoost classifier was trained using only these robust radiomic features. To ensure an unbiased and stable performance estimation, we utilized repeated 5-fold cross-validation (250 repetitions). This mitigates variance from single data splits and provides reliable confidence intervals for our multi-class metrics. To interpret this Final Robust Model, SHAP (SHapley Additive exPlanations) values were calculated to quantify the global importance and impact of each feature on the Final Robust Model’s decisions.

To compare the performance of different imaging models (T1-only, T2-only, and combined T1 and T2), the overall Accuracy and AUC were evaluated using the Wilcoxon signed-rank test. A two-sided *p*-value of <0.05 was considered statistically significant.

## 3. Results

The final study cohort included 332 patients distributed as follows: LMS (*n* = 67), MFS (*n* = 55), mLPS (*n* = 60), ddLPS (*n* = 33), UPS (*n* = 70), and MYX (*n* = 47). This distribution reflects a moderate class imbalance across subtypes, with certain categories such as dedifferentiated being less represented. This imbalance may influence model performance by favoring more prevalent classes. To mitigate this effect, all data splits were stratified by subtype. The lower extremity was the predominant tumor location for MFS (73%), mLPS (88%), and UPS (77%), whereas ddLPS was most frequent in the abdomen/pelvis (39%), and LMS was found in both regions (lower extremity 51%, abdomen/pelvis 31%). Tumor grade also varied significantly; G3 (high-grade) tumors were most common in UPS (80%) and ddLPS (61%), while mLPS frequently presented as G1 (low-grade) (38%). Table 1 shows patient demographics and clinical characteristics.

Inter-observer variability analysis on the 20% validation subset demonstrated excellent segmentation reproducibility, with a mean intra-class correlation coefficient (ICC) of 0.92 [29]. This high level of agreement validates the spatial robustness of the radiomic features selected for subsequent modeling.

PCA-weighted importance model demonstrated the strongest performance among the importance-based models (Table 2) and was selected for the Final Robust Model development. The PCA plot (Figure 3) reveals a tight, central cluster of runs surrounded by a small number of outliers, suggesting consistent performance for the core radiomic feature set. Furthermore, the frequency plot demonstrates a strong positive correlation between a radiomic feature’s Combined Score and its selection frequency. This relationship identifies a core subset of “Robust Radiomic Features” that exhibit both high consistency and strong performance, confirming their reliability in the model. Collectively, these results highlight the stability and predictive power of the selected radiomic features in the combined T1 and T2 models.

**Figure 4:**
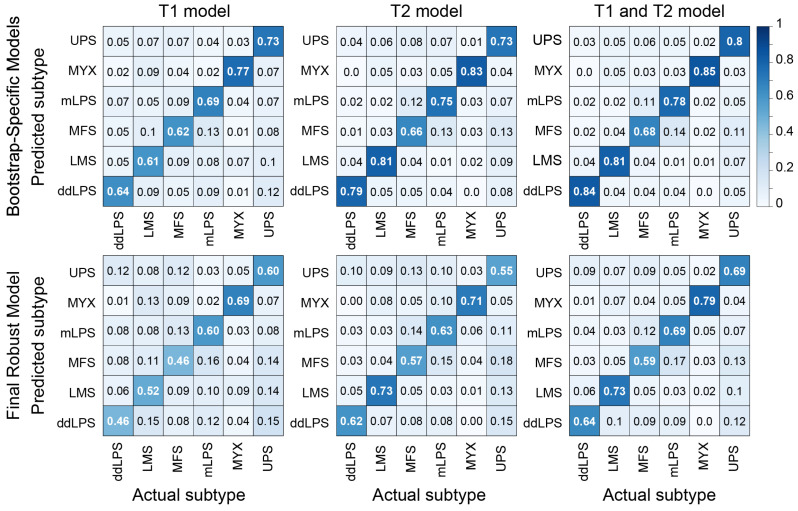
Confusion matrices for the classification of STS subtypes using T1, T2, and combined T1 and T2 MRI models. The top row displays average results from the bootstrap-specific models (aggregated from 250 random 70/30 splits). The bottom row shows the performance of the Final Robust Model, derived from repeated 5-fold cross-validation. In all matrices, diagonal elements represent accurate predictions.

**Figure 5:**
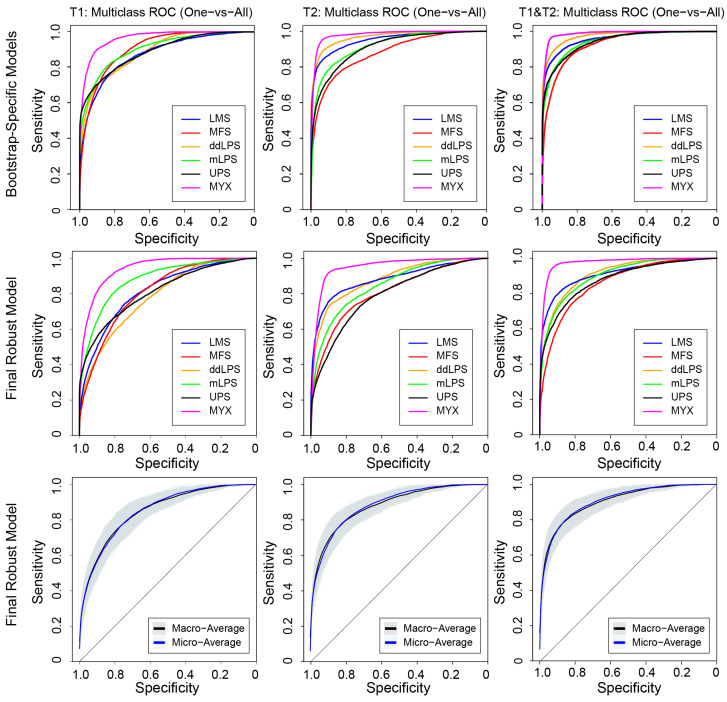
Multi-class ROC curves (One-vs-All) for the classification of STS subtypes using T1, T2, and combined T1 and T2 MRI models. The top row shows the performance of Bootstrap-Specific Models, while the middle row displays the performance of the Final Robust Model. The bottom row presents the macro- and micro-average multi-class ROC curves for the Final Robust Model across 250 bootstrap resamplings, with the shaded areas representing the 95% confidence intervals. All graphs plot Sensitivity against Specificity.

A comparison of models using different imaging inputs revealed that the combined T1 and T2 model consistently outperformed models built on T1-only or T2-only radiomic features. For the Final Robust Model, the combined approach yielded the highest correct classification rates across nearly all subtypes, ranging from 59% for MFS to 79% for MYX (Figure 4). The T2-only model performed better than the T1 model, with correct classification rates ranging from 57% for MFS to 73% for LMS. The T1-only model demonstrated the lowest performance, with corresponding rates ranging from a low of 46% (for both MFS and ddLPS) to a high of 69% for MYX. This performance trend was visually confirmed by the multi-class ROC curves (Figure 5), where the combined T1 and T2 model showed superior discrimination for all sarcoma subtypes.

The Final Robust Model combining T1 and T2 radiomic features achieved an overall accuracy of 0.68 ± 0.04 and an AUC of 0.92 ± 0.02 (Table 2). Bootstrap-specific models achieved higher peak metrics (Accuracy: 0.78 ± 0.03; AUC: 0.96 ± 0.01) using larger radiomic feature sets prior to robustness filtering. Across the 250 bootstrap iterations, the combined T1 and T2 model achieved significantly higher overall Accuracy and AUC compared to both the T1-only model (*p* < 0.001 for both metrics) and the T2-only model (*p* < 0.001 for both metrics). The Final Robust Model combining T1 and T2 features achieved significantly higher overall Accuracy compared to both the T1-only model (*p* = 5.12 × 10^−9^) and the T2-only model (*p* = 2.42 × 10^−7^). Similarly, the combined model demonstrated a significantly higher overall AUC than the T1-only (*p* = 1.74 × 10^−8^) and T2-only (*p* = 1.22 × 10^−7^) models. Furthermore, the T2-only model significantly outperformed the T1-only model in both Accuracy (*p* = 6.04 × 10^−7^) and AUC (*p* = 1.88 × 10^−5^). Notably, the macro-average and micro-average ROC curves are nearly entirely overlapping. This convergence indicates that the model maintains high discriminative power uniformly across all histologic subtypes, successfully mitigating bias toward the more prevalent classes in the cohort. Furthermore, the narrow 95% confidence intervals confirm the stability of the robust radiomic signature against variations in data splitting.

At the subtype level, balanced accuracy was highest for MYX (0.91 ± 0.05), ddLPS (0.84 ± 0.06), and LMS (0.83 ± 0.05) (Table 3). MFS showed the greatest classification ambiguity, with a correct classification rate of 59% and balanced accuracy of 0.76 ± 0.05. Misclassification of MFS occurred primarily as mLPS (17%) or UPS (13%). UPS was correctly classified in 69% of cases and most frequently misclassified as MFS (9%) or mLPS (9%). LMS was correctly classified in 73% of cases, with misclassification primarily as UPS (10%) (Figure 4).

**Figure 6:**
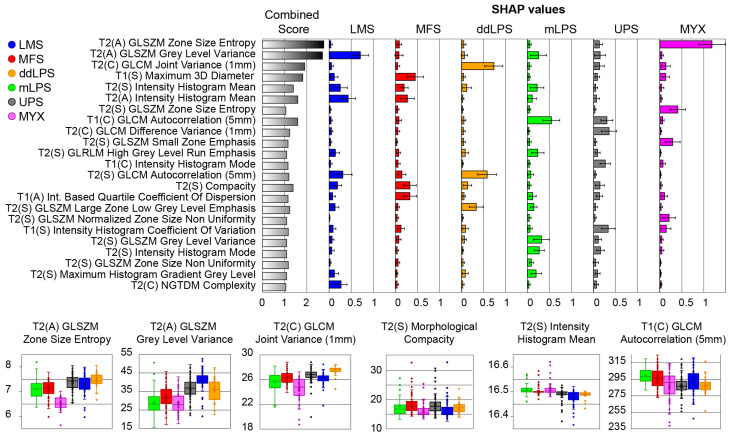
SHAP values and box plots of top radiomic features for STS subtype classification using combined T1 and T2 models. The left panel shows the “Combined Score” and individual SHAP values for each subtype for various radiomic features. The bottom row presents box plots illustrating the distribution of six key radiomic features across the different STS subtypes.

SHAP analysis identified a limited number of highly robust features driving subtype classification (Figure 6). As detailed in the Discussion, these top-ranking mathematical features closely align with the known macroscopic and histopathological characteristics of the respective tumors. For MYX, T2 GLSZM Zone Size Entropy was among the most influential features. For LMS, T2 GLSZM Gray-Level Variance contributed strongly to model predictions. For ddLPS, T2 GLCM Joint Variance at 1 mm distance demonstrated high importance. Several features showed overlapping distributions across MFS, UPS, and mLPS.

## 4. Discussion

Our findings demonstrate the feasibility of radiomics-based MRI analysis for histologic subtype classification of soft tissue sarcomas. The Final Robust Model combining T1- and T2-weighted sequences achieved an overall classification accuracy of 68% and an AUC of 0.92. The apparent discrepancy between the moderate overall accuracy and the high AUC can be explained by the mathematical differences between these metrics in a multi-class setting. Our multi-class AUC (calculated via a One-vs-All approach) evaluates the model’s ability to discriminate a specific subtype from all others across all possible classification thresholds, reflecting excellent underlying probability ranking. In contrast, overall accuracy depends on a single, fixed decision threshold. In a dataset with moderate class imbalance and overlapping radiomic profiles (such as the shared myxoid components in MFS and mLPS), this single threshold is highly sensitive to hard misclassifications, leading to a lower accuracy despite the high discriminative power captured by the AUC. While this performance remains below that of expert sarcoma pathology, it compares favorably with reported core needle biopsy accuracies ranging between 60% and 80% [37,38]. By capturing quantitative information from the entire tumor volume, radiomics may better reflect spatial heterogeneity than sampling-based approaches [39,40]. Consequently, integrating whole-tumor radiomic signatures with traditional core needle biopsies could help guide needle placement to the most representative or aggressive tumor regions, ultimately improving overall diagnostic confidence.

A key finding was the balance between model performance and generalizability. Although bootstrap-specific models achieved higher peak accuracy, the Final Robust Model relied only on features consistently selected across 250 resampling iterations. This deliberate constraint reduced maximal performance but enhanced stability and portability, limiting the risk of overfitting in the context of relatively limited sample sizes.

The best results were consistently achieved using a combination of T1- and T2-weighted MRI sequences, underscoring the complementary biological information provided by multiparametric imaging. While T2-weighted features captured substantial intratumoral heterogeneity, the addition of contrast-enhanced T1-weighted information further improved discrimination, suggesting that vascular and enhancement-related characteristics contribute meaningfully to subtype differentiation.

The high classification performance in Balanced Accuracy for MYX (0.91 ± 0.05), ddLPS (0.84 ± 0.06), and LMS (0.83 ± 0.05) represents one of the most clinically relevant findings of this study. These tumors often exhibit distinct morphological and textural features on MRI that are amenable to radiomic quantification. This observation is consistent with prior studies showing the discriminative power of imaging phenotypes in sarcoma classification [18,19,41].

By contrast, the persistent challenge in differentiating mLPS (0.78 ± 0.05), UPS (0.77 ± 0.05), and MFS (0.76 ± 0.05) underscores the intrinsic limitations of both imaging- and pathology-based methods in resolving histologic ambiguity. In particular, MFS and mLPS may both present with prominent myxoid stroma and hyperintense T2 signal, leading to confusion on MRI [42]. Overlapping radiomic distributions among these subtypes likely reflect shared architectural complexity and myxoid components, explaining the observed classification ambiguity.

Notably, intramuscular myxoma, the only benign lesion in our cohort, was accurately classified with high sensitivity (0.86 ± 0.10) and specificity (0.95 ± 0.02). This finding suggests that radiomics may also contribute to benign–malignant discrimination in selected cases and could potentially support non-invasive triage in patients with imaging features suggestive of benignity. Compared to prior radiomics studies in soft tissue sarcomas, most of which have focused on binary classification tasks (e.g., benign versus malignant) or tumor grading, our study addresses the more complex problem of multi-class histologic subtype differentiation. For example, in a study focused on tumor grading, Peeken et al. demonstrated the differentiation of high- and low-grade soft tissue sarcomas (STS) using T2FS and T1FSGd MRI sequences; their T2FS, T1FSGd, and combined models achieved predictive performances with areas under the receiver operating characteristic curve (AUCs) of 0.78, 0.69, and 0.76, respectively, on an independent validation set of 103 patients [19].

SHAP analysis provided insight into the radiomic patterns underlying these classifications. For intramuscular myxoma, low T2 GLSZM Zone Size Entropy was a dominant predictive feature. This likely reflects the known homogeneous myxoid stroma of myxomas, which typically produce uniform high T2 signal intensity. In leiomyosarcoma, high T2 GLSZM Gray-Level Variance was strongly associated with model predictions, consistent with the complex internal architecture of these tumors, often composed of necrosis, hemorrhage, cystic change, and solid components. Similarly, higher T2 GLCM Joint Variance at a 1 mm spatial distance in dedifferentiated liposarcoma suggests increased local texture heterogeneity, potentially corresponding to abrupt microstructural transitions within the tumor. Conversely, overlapping distributions of features such as T2 intensity histogram mean, morphological compactness, and T1 GLCM autocorrelation were observed among myxofibrosarcoma, undifferentiated pleomorphic sarcoma, and myxoid liposarcoma. These shared radiomic characteristics likely reflect common structural complexity and myxoid or heterogeneous components across these subtypes, explaining the observed classification ambiguity.

Overall, these findings enhance the clinical interpretability of the model, supporting the interpretation of radiomic features as quantitative surrogates of tumor microstructure and heterogeneity. By directly correlating abstract textural metrics with known histopathological realities (such as necrosis in LMS or homogeneity in myxomas), the model captures variations in tissue composition and spatial organization that are not readily appreciable on qualitative MRI assessment.

From a clinical perspective, this model could be integrated into the radiological workflow as a decision-support tool applied to pre-treatment MRI examinations. Radiomic analysis could provide probabilistic predictions of histologic subtype prior to biopsy, assisting radiologists in refining differential diagnoses. In addition, such information could help guide biopsy targeting toward the most representative or heterogeneous tumor regions, particularly in large or complex lesions. In selected cases, radiomic signatures suggestive of benign lesions, such as intramuscular myxoma, may also contribute to non-invasive triage. Beyond initial assessment, applying these quantitative models longitudinally as delta radiomics could provide a critical tool for monitoring tumor response to neoadjuvant therapy [6,36]. While not intended to replace histopathological diagnosis, this approach may complement conventional imaging interpretation and improve diagnostic confidence in specialized sarcoma centers.

The strength of our findings is supported by the study design and cohort size. We included 332 patients across six clinically relevant histologic subtypes, which compares favorably with prior MRI-based radiomics studies in soft tissue sarcoma that have typically included smaller cohorts and often focused on binary classification or tumor grading tasks rather than multi-class subtype differentiation (ref to add). In addition to cohort size, our approach incorporated volumetric segmentation, multiparametric MRI inputs, and a rigorous bootstrap-based validation strategy with nested cross-validation to mitigate overfitting and enhance reproducibility.

Nonetheless, this study has several limitations that must be acknowledged. First, the study is retrospective, single-institution nature warrants caution regarding generalizability until external validation is performed. Second, the significant variation in tumor location across subtypes, a known clinical pattern, presents a challenging classification scenario that may require future anatomy-aware models to fully resolve. Third, there is an imbalance in subtype representation, with certain classes (e.g., dedifferentiated liposarcoma) being less represented than others. Class imbalance may bias model training toward majority classes and reduce performance for rarer subtypes. To mitigate this, all splits were stratified by subtype and balanced accuracy was used to account for unequal class sizes. Fourth, manual segmentation, while ensuring high-quality tumor delineation, is time-consuming and not scalable for routine clinical implementation. The sensitivity of radiomic features to segmentation differences may also impact model generalizability. Future work should explore semi-automated or fully automated AI-based segmentation approaches to improve efficiency and reproducibility. Fifth, only six mesenchymal tumor subtypes were included, which do not encompass the full histologic diversity of STS. Sixth, variability in MRI acquisition protocols across scanners and time periods may have influenced radiomic feature extraction, although this reflects real-world clinical conditions. Seventh, while XGBoost was selected for its robustness in high-dimensional settings and regularization capabilities, this study was limited to a single classifier. Future research should prioritize comparing performance across different models such as Random Forest (RF) or Support Vector Machines (SVM), as well as exploring ensemble methods or mixture of expert approaches to further enhance diagnostic accuracy.

The clinical utility of radiomics extends far beyond initial classification, holding significant promise for personalizing sarcoma care by moving beyond initial classification [43,44]. Future work should focus on creating more comprehensive prognostic tools by integrating radiomic data with histologic, genomic, and clinical features [45,46]. The development of subtype-specific delta radiomics models [36,47], which analyze feature changes after neoadjuvant therapy, is also a critical next step for monitoring treatment response where traditional size-based metrics are insufficient [48,49]. Ultimately, large, multi-center collaborations are essential to build robust, generalizable models that can improve diagnostic accuracy and patient triage, thereby enhancing clinical workflow and patient care.

## 5. Conclusions

Applying radiomics to pre-treatment MRI offers a robust, non-invasive approach to classifying soft tissue sarcoma subtypes. This method directly addresses a major challenge in diagnosing and managing these rare tumors, as it enhances diagnostic confidence and informs treatment strategies. The model presented in this study represents a vital proof-of-concept, establishing the foundation for future advancements in sarcoma imaging through integrated, multi-modal models for personalized care and improved prognostication.

## Figures and Tables

**Table 1:** Patient Characteristics.

Histotype	LMS	MFS	mLPS	ddLPS	UPS	MYX
**Subject count (332 total)**						
**Female**	40 (60%)	25 (45%)	28 (47%)	11 (33%)	42 (60%)	34 (72%)
**Male**	27 (40%)	30 (55%)	32 (63%)	22 (67%)	28 (40%)	13 (28%)
**Total**	67	55	60	33	70	47
**Age**						
**Average ± SD**	59 ± 15	66 ± 12.5	47 ± 21.5	62 ± 11	62 ± 16	54 ± 17
**Grade**						
**G1**	6 (9%)	7 (14%)	23 (38%)	2 (6%)	0 (0%)	0 (0%)
**G2**	17 (25%)	20 (36%)	11 (18%)	9 (27%)	13 (19%)	0 (0%)
**G3**	34 (51%)	25 (45%)	10 (17%)	20 (61%)	56 (80%)	0 (0%)
**N/A**	10 (15%)	3 (5%)	16 (27%)	2 (6%)	1 (1%)	47 (100%)
**Tumor location**						
**Upper extremity**	9 (13%)	12 (22%)	1 (2%)	3 (9%)	8 (11%)	9 (19%)
**Lower extremity**	34 (51%)	40 (73%)	53 (88%)	16 (48%)	54 (77%)	33 (70%)
**Abdomen/Pelvis**	21 (31%)	1 (2%)	5 (8%)	13 (39%)	5 (7%)	5 (11%)
**Trunk**	1 (1%)	2 (4%)	1 (2%)	1 (3%)	3 (4%)	0 (0%)
**Other**	2 (3%)	0 (0%)	0 (0%)	0 (0%)	0 (0%)	0 (0%)
**Metastasis**						
**Yes**	38 (57%)	11 (20%)	13 (22%)	14 (41%)	29 (42%)	0 (0%)
**No**	29 (43%)	44 (80%)	47 (78%)	19 (59%)	41 (58%)	0 (0%)
**Not available**	0 (0%)	0 (0%)	0 (0%)	0 (0%)	0 (0%)	47 (100%)

**Table 2:** Overall Model Performance Metrics.

T1 & T2	PCA-Weighted Importance	Mean Importance	Median Importance	Frequency
**Bootstrap-Specific Models (all radiomic features)**				
**Accuracy**	0.78+/−0.03	0.77+/−0.03	0.77+/−0.03	0.77+/−0.04
**Kappa**	0.72+/−0.04	0.72+/−0.04	0.72+/−0.03	0.72+/−0.04
**AUC**	0.96+/−0.01	0.96+/−0.01	0.95+/−0.01	0.95+/−0.01
**Final Robust Model (robust radiomic features)**				
**Accuracy**	0.68+/−0.04	0.66+/−0.04	0.67+/−0.04	0.67+/−0.03
**Kappa**	0.61+/−0.05	0.59+/−0.05	0.60+/−0.05	0.60+/−0.04
**AUC**	0.92+/−0.02	0.91+/−0.02	0.91+/−0.02	0.91+/−0.02

**Table 3:** Subtype-Specific Classification Performance.

	LMS	MFS	ddLPS	mLPS	UPS	MYX
**T1 & T2 Bootstrap-Specific Models (all features)**						
**Sensitivity**	0.83 ± 0.07	0.66 ± 0.12	0.82 ± 0.12	0.73 ± 0.09	0.75 ± 0.08	0.89 ± 0.09
**Specificity**	0.96 ± 0.02	0.92 ± 0.03	0.98 ± 0.01	0.94 ± 0.02	0.94 ± 0.03	0.97 ± 0.02
**PPV**	0.83 ± 0.08	0.65 ± 0.13	0.86 ± 0.14	0.75 ± 0.09	0.77 ± 0.08	0.83 ± 0.09
**NPV**	0.96 ± 0.02	0.93 ± 0.02	0.98 ± 0.01	0.94 ± 0.02	0.93 ± 0.02	0.98 ± 0.02
**Balanced Accuracy**	0.90 ± 0.04	0.79 ± 0.06	0.90 ± 0.06	0.84 ± 0.04	0.84 ± 0.04	0.93 ± 0.04
**F1 Class**	0.83 ± 0.05	0.64 ± 0.10	0.83 ± 0.10	0.73 ± 0.07	0.76 ± 0.06	0.85 ± 0.05
**T1 & T2 Final Robust Model (robust features)**						
**Sensitivity**	0.72 ± 0.1	0.57 ± 0.1	0.7 ± 0.12	0.63 ± 0.09	0.63 ± 0.11	0.86 ± 0.1
**Specificity**	0.94 ± 0.03	0.92 ± 0.03	0.95 ± 0.02	0.93 ± 0.03	0.91 ± 0.04	0.95 ± 0.02
**PPV**	0.73 ± 0.1	0.62 ± 0.13	0.64 ± 0.12	0.68 ± 0.11	0.66 ± 0.1	0.76 ± 0.1
**NPV**	0.94 ± 0.02	0.91 ± 0.03	0.96 ± 0.02	0.92 ± 0.03	0.90 ± 0.03	0.97 ± 0.02
**Balanced Accuracy**	0.83 ± 0.05	0.76 ± 0.05	0.84 ± 0.06	0.78 ± 0.05	0.77 ± 0.05	0.91 ± 0.05
**F1 Class**	0.72 ± 0.07	0.58 ± 0.08	0.66 ± 0.09	0.65 ± 0.08	0.64 ± 0.07	0.80 ± 0.07

## Data Availability

The original contributions presented in this study are included in the article. Further inquiries can be directed to the corresponding author.

## Supplementary Materials

The following supporting information can be downloaded at: https://www.mdpi.com/article/10.3390/cancers18101667/s1, Paragraph S1: MRI Acquisition Parameters and MRI Sequence Distribution: title; Table S1: MRI Acquisition Parameters, Vendor Distribution, Field Strength, by STS subtype; Figure S1: Scatter plot showing the association between radiomic features and tumor size. The red line represents the linear mixed-effects model used to impute missing radiomic features.

## Abbreviations

The following abbreviations are used in this manuscript:

MRI	Magnetic Resonance Imaging
T1 FS CE	T1-Weighted Fat-Saturated Contrast-Enhanced
T2 FS	T2-Weighted Fat-Saturated
HIPAA	Health Insurance Portability And Accountability Act
IRB	Institutional Review Board
PACS	Picture Archiving And Communication System
EDW	Electronic Data Warehouse
STS	Soft Tissue Sarcoma
LMS	Leiomyosarcoma
MFS	Myxofibrosarcoma
mLPS	Myxoid Liposarcoma
ddLPS	Dedifferentiated Liposarcoma
UPS	Undifferentiated Pleomorphic Sarcoma
MYX	Intramuscular Myxoma
AUC	Area Under the Curve
GLCM	Gray-Level Co-Occurrence Matrix
GLRLM	Gray-Level Run-Length Matrix
GLSZM	Gray-Level Size Zone Matrix
NGTDM	Neighboring Gray Tone Difference Matrix
PCA	Principal Component Analysis
NCV	Nested Cross-Validation
SHAP	Shapley Additive Explanations
