# Supplementary material for: A Predictive MRI Radiomics Model for Histologic Differentiation in Soft Tissue Sarcomas
Source: Cancers (Basel). 2026 May 21;18(10):1667. doi: 10.3390/cancers18101667 (PMC13204288; doi:10.3390/cancers18101667)
Supplement: Supplementary file 1 [file cancers-18-01667-s001.zip › cancers-4285336-supplementary.pdf]

Paragraph S1: MRI Acquisition Parameters and MRI Sequence Distribution

The available MRI sequences for analysis included 281 T1 axial, 254 T1 coronal, 248 T1 sagittal, 265 T2 axial, 247 T2 coronal, and 220 T2 sagittal scans. Of all subjects, 89% had T1 scans, 92% had T2 scans, and 80% had both T1 and T2 scans. Among all subjects, approximately 55% had all three T1-weighted sequences with at least one T2 sequence also available, and 42% had all three T2-weighted sequences with at least one T1 sequence also available. Additionally, 25% of subjects had only two T1-weighted scans, and 37% had only two T2-weighted scans with at least one other sequence available. Only 12 subjects had one T1 and one T2 scan, and 20 subjects had just one scan overall.

Table S1: MRI Acquisition Parameters, Vendor Distribution, Field Strength, by STS subtype

Subtypes	LMS	MFS	MYX	mLPS	UPS	ddLPS
**T1CE Parameters**						
**TE (ms)**	9.5	10.6	9.8	10.8	10.4	6.6
**TR (ms)**	530	614	623	535	587	378
**Pixel spacing (mm)**	0.73	0.58	0.71	0.69	0.74	0.85
**Slice thickness (mm)**	4.56	4.9	4.31	5.19	5.03	4.91
**Rows**	380	395	405	384	368	382
**Columns**	389	402	424	386	372	390
**T2 Parameters**						
**TE (ms)**	63.9	59.6	56.3	56.1	59.4	68.9
**TR (ms)**	4973	4611	4192	4530	4639	4673
**Pixel spacing (mm)**	0.65	0.62	0.67	0.76	0.73	0.82
**Slice thickness (mm)**	4.75	4.86	4.45	5.19	5.28	5.14
**Rows**	394	388	406	359	367	386
**Columns**	399	380	418	360	364	397
**Vendor Distribution (%)**						
**GE**	40%	31%	21%	28%	26%	45%
**Siemens**	52%	51%	77%	40%	60%	48%
**Philips**	4%	9%	2%	8%	6%	0%
**Hitachi**	1%	4%	0%	3%	4%	0%
**Toshiba**	1%	5%	0%	3%	4%	3%
**Field Strength Distribution (%)**						
**1.5T**	68.7%	69.1%	51.1%	81.7%	72.9%	81.8%
**3T**	26.9%	27.3%	48.9%	11.7%	22.9%	18.2%
**Other (less than 1.5T)**	4.5%	3.6%	0.0%	6.7%	4.3%	0.0%

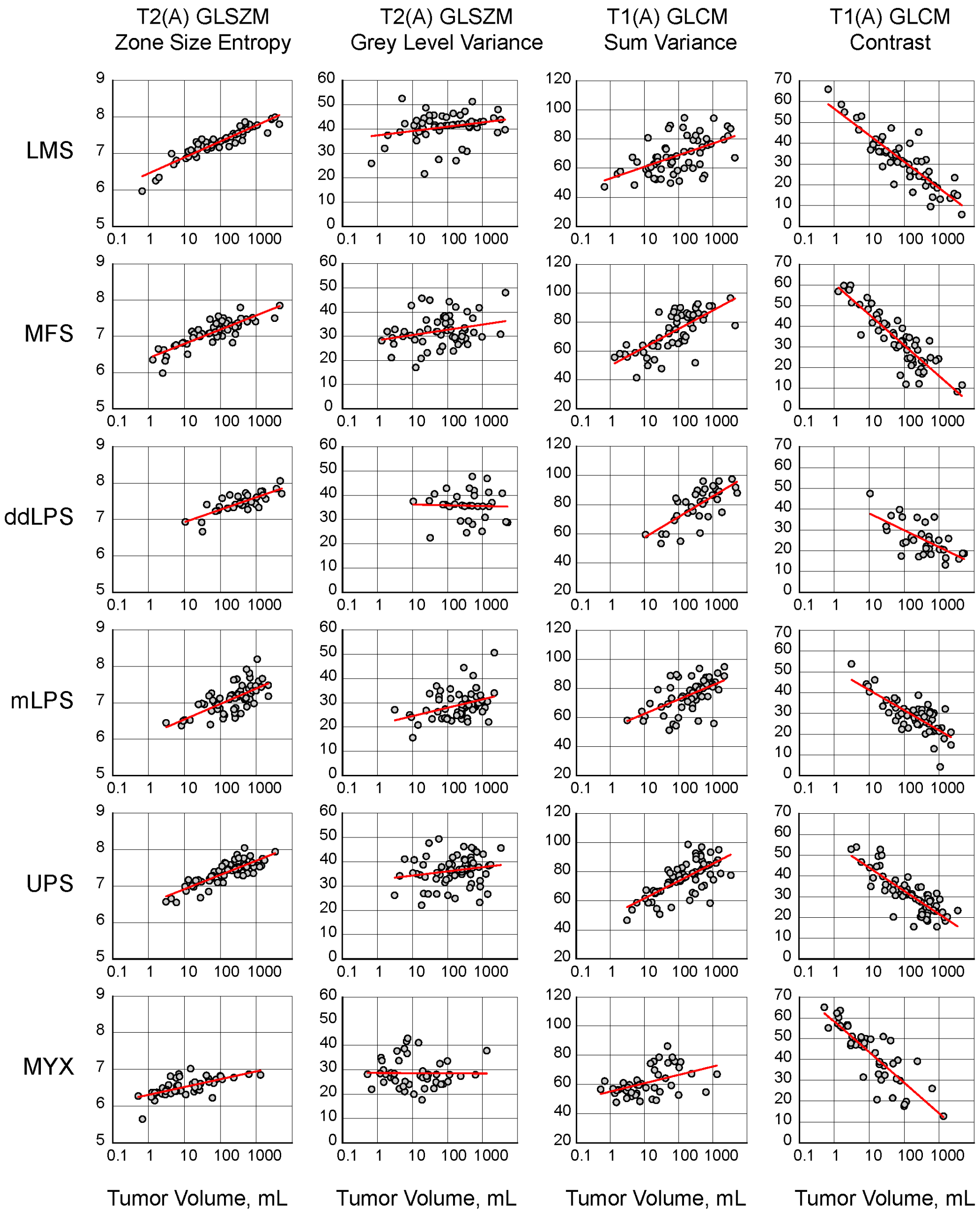

Figure S1: Scatter plot showing the association between radiomic features and tumor size. The red line represents the linear mixed-effects model used to impute missing radiomic features.
